# Supplementary material for: The Childhood Resilience Study: Resilience and emotional and behavioural wellbeing experienced by Australian Aboriginal and Torres Strait Islander boys and girls aged 5–9 years
Source: PLoS One. 2024 Apr 16;19(4):e0301620. doi: 10.1371/journal.pone.0301620 (PMC11020770; doi:10.1371/journal.pone.0301620)
Supplement: S1 Checklist — (DOCX) [file pone.0301620.s001.docx]

Inclusivity in global research

PLOS’ policy on inclusivity in global research aims to improve transparency in the reporting of research performed outside of researchers’ own country or community and ensures that PLOS publications reporting global research adhere to high standards for research ethics and authorship. Authors of relevant research articles may be asked to complete the questionnaire below, which outlines ethical, cultural, and scientific considerations specific to inclusivity in global research. This questionnaire may be requested when researchers have travelled to a different country to conduct research, if research uses samples collected in another country, research with Indigenous populations or their lands, or if research is on cultural artefacts. Researchers travelling to another country solely to use laboratory equipment will not normally be required to complete the questionnaire. However, the questionnaire can be requested at the journal’s discretion for any submission – if you have been requested to complete this questionnaire by the PLOS journal you submitted to, please do so.

Please complete the questionnaire below and include this as a Supporting Information file with your manuscript. Note that if your paper is accepted for publication, this checklist will be published with your article in the supporting information files. Please ensure that you reference the checklist in the main body of your manuscript. We suggest adding a subsection ‘Inclusivity in global research’ to your Methods section and adding the following sentence: “Additional information regarding the ethical, cultural, and scientific considerations specific to inclusivity in global research is included in the Supporting Information (SX Checklist)”

The questions have been designed to be applicable to a wide range of study types, and there are subsections for both human subjects research and non-human subjects research. If any of the questions are not relevant to your research please mark them as “N/A” as appropriate.

**Ethical considerations, permits and authorship**

*This section is applicable to all research types.*

Provide details as to who granted permissions and/or consent for the study to take place in the Methods section of your manuscript. This should include the names of **all** ethics boards, governmental organizations, community leaders or other bodies that provided approval for the study. If individuals provided approval refer to these people by their role or title but do not list their name(s).

Reported on page number: 5

If there were any deviations from the study protocol after approval was obtained please provide details of these changes in the Methods section of your manuscript.
Did this study involve local collaborators that are residents of the country where the research was conducted or members of the community studied? If you do not have any authors from said communities, please provide an explanation for this below.

Reported on page number: N/A

This research was conducted in the country now called Australia, where the sovereignty of Aboriginal and Torres Strait Islander (First Nations) tradional lands has never been ceded.

The study was undertaken undertaken in partnership with the Aboriginal Health Council of South Australia (peak body for Aboriginal Community Controlled Health Organisations in this state), with guidance of an Aboriginal Governance Group auspiced by the Council.

All contact with Aboriginal and Torres Strait Islander participants was undertaken by Aboriginal researchers residing on Kaurna Country and/or local communities participating in the research. In addition, relationships were built with local Aboriginal Community Controlled Organisations to support entry into communities and ongoing community engagement.

Aboriginal authors on this paper are Arwen Nikolof (who led the team of Aboriginal researchers involved in data collection), Karen Glover and Cathy Leane (members of the Aboriginal Governance Group and Study Investigator team) and Graham Gee (Aboriginal researcher and member of study investigator team).

Everyone listed as an author should meet PLOS’ criteria for authorship and all individuals who meet these criteria should be included in the author byline, rather than the acknowledgements. For further information please see the journal’s Authorship Policy.

**Human subjects research (e.g. health research, medical research, cross-cultural psychology)**

Did you obtain written informed consent from a representative of the local community or region before the research took place? How did you establish who speaks for the community? Details of written informed consent obtained from study participants should be reported separately in the Methods section of your manuscript.

The Childhood Resilience Study originated from consultations with Aboriginal communities in urban, regional and remote areas of South Australia undertaken to inform design of the Aboriginal Families Study. Community members wanted to understand why some children were doing well, while others in similar situations were not seeming to do as well. These consultations were undertaken in partnership with the Aboriginal Health Council of South Australia, with guidance from the study’s Aboriginal Governance Group.

The project has approval from the Aboriginal Health Research Ethics Committee in South Australia, the governing ethics committee for Aboriginal health research in South Australia. This partnership meant that the Aboriginal Health Council through its CEO and Deputy CEO provided advice as members of and in the Aboriginal Governing Group meetings about who the appropriate local people were to contact. Aboriginal researchers followed up, often knowing the local person. Where the local person was unknown to the researcher, the Council would make an introduction. Arrangements were always made via phone and email prior to visiting at a time identified by the local community.

In addition, the Murdoch Children’s Research Institute (administering institution) has a partnership agreement with the Aboriginal Health Council of South Australia covering governance arrangements, including community consent for the project.

How did members of the local community provide input on the aims of the research investigation, its methodology, and its anticipated outcome(s)?

Local communities across urban, regional and remote areas of South Australia were invited to participate in community consultations over an 18 month period (2007-2008). These consultations were undertaken by two Aboriginal researchers, with guidance from the Aboriginal Governance Group (AGG). Feedback was sought on the focus of the research, culturally appropriate ways for the research team to engage Aboriginal families, and outcomes that communities wanted from the research. AGG members supported the research team to interpret community feedback and worked with the research team to co-design the study protocols for the Aboriginal Families Study (2008-2010) and the Childhood Resilience Study (2012-2013).

When engaging with the local community, how did you ensure that the informed consent documents and other materials could be understood by local stakeholders?

Aboriginal researchers explained what the study was about, why the research was being done, and what was involved in taking part. They ensured that families approached to take part understood all aspects of the consent materials and participant information sheet.

Families were encouraged to ask questions before deciding to participate. Parents provided written or verbal consent for themselves and/or their child to participate. Where verbal consent was provided, the researcher completed a written consent form confirming that active verbal consent for participation had been gained. Children aged 7 or older were invited to provide verbal or written assent to self-completing the CRQ-C.

Parents/caregivers and the researcher remained present when the child was completing the survey so that support or assistance was available where necessary.

Will the findings of the research be made available in an understandable format to stakeholders in the community where the study was conducted (e.g. via a presentation, summary report, copies of publications, etc.)? Please provide details of how this will be achieved.

Findings from the study will be shared with communities where the research was conducted via:

- community accountability forums held by the Aboriginal Communities and Families Health Research Alliance
- the Stronger Futures Centre of Research Excellence website (strongerfutures.org.au)
- the Aboriginal Health Council of South Australia newsletter.

We are currently working on a brief community resource for distribution via these pathways. The Aboriginal Governance Group has oversight of all community feedback activities.

**Non-human subjects research using specimens/ animals collected as part of the study, or those housed in archival collections. Examples include archaeology, paleontology, botany and zoology.**

Did the permission you obtained from a local authority to perform the study include an agreement on access to outputs and benefit sharing? This may include procedures to enable fair distribution of the benefits and resources arising from the research performed. Please include any details of Prior Informed Consent and Benefit Sharing Agreements obtained. These may be required by field-specific regulations, for example the Convention on Biological Diversity (CBD) and the associated Nagoya Protocol.

Not applicable

If the material used in your study was imported, please A) provide the year it was imported and B) indicate whether permits were obtained to import/export the materials used, C) provide details of any permits obtained. If this information is not available, please indicate this.

Not applicable

If you used archival specimens, please state how the material used in your study was acquired by the institute it is held in and provide details of any permits obtained for the original excavations/ sample collection. If this information is not available, please indicate this.

Not applicable

How was the potential cultural significance of the materials collected in your study to local communities considered in your research design? Were Indigenous peoples and/or local researchers and institutions involved with archaeological excavations / collection of specimens? If so, please provide a description of their involvement.

Not applicable

If your manuscript includes photographs of human remains please indicate whether authors obtained permission from descendants or affiliated cultural communities to do so.

Not applicable
