# Supplementary material for: The Childhood Resilience Study: Resilience and emotional and behavioural wellbeing experienced by Australian Aboriginal and Torres Strait Islander boys and girls aged 5–9 years
Source: PLoS One. 2024 Apr 16;19(4):e0301620. doi: 10.1371/journal.pone.0301620 (PMC11020770; doi:10.1371/journal.pone.0301620)
Supplement: S1 Table — (DOCX) [file pone.0301620.s002.docx]

**S1 Table. Aboriginal Families Study sample, family characteristics by child gender (n=231).**

|  | Child gender | | Logistic Regression | |
| --- | --- | --- | --- | --- |
|  | Boy | Girl | (Odds of being a girl) | |
|  | n (%) | n (%) | OR [95%CI] | p-value |
| **All respondents (n=231)** |  |  |  |  |
| Child Age |  |  |  |  |
| 5-6 years | 68 (52.7) | 61 (47.3) | 1.0 [ref] |  |
| 7-9 years | 61 (59.8) | 41 (40.2) | 0.7 [0.4, 1.3] | 0.282 |
| Place of residence (ABS 2016) |  |  |  |  |
| Urban | 61 (58.1) | 44 (41.9) | 1.0 [ref] |  |
| Regional | 45 (55.6) | 36 (44.4) | 1.1 [0.6, 2.0] | 0.729 |
| Remote | 23 (51.1) | 22 (48.9) | 1.3 [0.7, 2.7] | 0.430 |
| Mother is Aboriginal and/or Torres Strait Islander |  |  |  |  |
| No | 16 (66.7) | 8 (33.3) | 1.0 [ref] |  |
| Yes | 113 (54.6) | 94 (45.4) | 1.7 [0.7, 4.1] | 0.263 |
| Father is Aboriginal and/or Torres Strait Islander |  |  |  |  |
| No | 30 (55.6) | 24 (44.4) | 1.0 [ref] |  |
| Yes | 98 (56.0) | 77 (44.0) | 1.0 [0.5, 1.8] | 0.954 |
| Child living with: |  |  |  |  |
| Mother | 114 (54.8) | 94 (45.2) | 1.0 [ref] |  |
| Other family (not mother) | 13 (65.0) | 7 (35.0) | 0.7 [0.3, 1.7] | 0.384 |
| Foster care | 2 (66.7) | 1 (33.3) | 0.6 [0.1, 6.8] | 0.685 |
| Adults in household (past month) |  |  |  |  |
| One adult | 44 (60.3) | 29 (39.7) | 0.9 [0.5, 1.6] | 0.696 |
| Two adults | 66 (57.4) | 49 (42.6) | 1.0 [ref] |  |
| 3+ adults | 16 (41.0) | 23 (59.0) | 1.9 [0.9, 4.0] | 0.079 |
| OWN children living with mother/primary caregiver |  |  |  |  |
| None | 12 (70.6) | 5 (29.4) | 0.5 [0.2, 1.7] | 0.284 |
| 1-2 | 52 (56.5) | 40 (43.5) | 1.0 [ref] |  |
| 3-4 | 48 (51.1) | 46 (48.9) | 1.2 [0.7, 2.2] | 0.456 |
| 5+ | 17 (60.7) | 11 (39.3) | 0.8 [0.4, 2.0] | 0.695 |
| OTHER children living with mother/primary caregiver |  |  |  |  |
| None | 102 (56.0) | 80 (44.0) | 1.0 [ref] |  |
| 1-2 | 21 (60.0) | 14 (40.0) | 0.9 [0.4, 1.8] | 0.666 |
| 3+ | 6 (42.9) | 8 (57.1) | 1.7 [0.6, 5.1] | 0.344 |
| Age of mother/primary caregiver |  |  |  |  |
| 20-24 years | 5 (41.7) | 7 (58.3) | 2.3 [0.7, 7.9] | 0.194 |
| 25-29 years | 38 (50.7) | 37 (49.3) | 1.6 [0.8, 3.1] | 0.170 |
| 30-34 years | 44 (62.0) | 27 (38.0) | 1.0 [ref] |  |
| 35+ years | 42 (57.5) | 31 (42.5) | 1.2 [0.6, 2.3] | 0.587 |
| Total | 129 (55·8) | 102 (44·2) |  |  |
| **Mothers in cohort (n=208)** |  |  |  |  |
| Mothers age at birth of study child |  |  |  |  |
| 15-19 years | 17 (53.1) | 15 (46.9) | 1.1 [0.5, 2.4] | 0.866 |
| 20-24 years | 45 (54.9) | 37 (45.1) | 0.8 [0.4, 1.5] | 0.441 |
| 25-29 years | 38 (61.3) | 24 (38.7) | 1.0 [ref] |  |
| 30+ years | 29 (52.7) | 26 (47.3) | 1.1 [0.5, 2.2] | 0.804 |
| Relationship status |  |  |  |  |
| Living with partner | 45 (49.5) | 46 (50.5) | 1.0 [ref] |  |
| Single | 57 (60.0) | 38 (40.0) | 0.7 [0.4, 1.2] | 0.149 |
| In a relationship but not living together | 11 (52.4) | 10 (47.6) | 0.9 [0.3, 2.3] | 0.809 |
| Highest level education |  |  |  |  |
| Year 10 or less | 58 (56.9) | 44 (43.1) | 1.0 [ref] |  |
| Completed Year 12 | 17 (54.8) | 14 (45.2) | 1.1 [0.5, 2.4] | 0.842 |
| Certificate/Diploma | 28 (50.0) | 28 (50.0) | 1.3 [0.7, 2.5] | 0.408 |
| Degree | 7 (46.7) | 8 (53.3) | 1.5 [0.5, 4.5] | 0.460 |
| Health care concession card |  |  |  |  |
| No | 22 (45.8) | 26 (54.2) | 1.0 [ref] |  |
| Yes | 92 (57.5) | 68 (42.5) | 0.6 [0.3, 1.2] | 0.156 |
| Currently employed |  |  |  |  |
| Yes | 39 (50.0) | 39 (50.0) | 1.0 [ref] |  |
| No | 74 (57.4) | 55 (42.6) | 0.7 [0.4, 1.3] | 0.303 |
| Currently Studying |  |  |  |  |
| Yes | 20 (55.6) | 16 (44.4) | 1.0 [ref] |  |
| No | 94 (54.7) | 78 (45.3) | 1.0 [0.5, 2.1] | 0.921 |
| Total | 114 (54.8) | 94 (45.2) |  |  |
